# Scientific Items

**Published:** 1879-07-20

**Authors:** 


### SCIENTIFIC ITEMS.

**A Voltaic Pencil.**—*Le Technologiste* gives the following description of an instrument which seems to be an improvement on Edison's electric pen : It is to Mr. Bellet, a Parisian, that we owe the invention of the voltaic pencil, which perforates the paper in the same manner that it is pierced by Edison's electric pen, but without the intervention of the needle vibrated by a little electro-motor. Instead of actuating a needle as in the ordinary pricking machines, used by those who design laces, embroideries, etc., the electric current itself passes through the paper; for, as is well known, the lead of a pencil is a good conductor. This arrangement is advantageous in that the artist sees the traces of his work, his method of working being in no way different from the one which he habitually uses. But more than this, by means of this voltaic pencil, so skillfully perfected by its inventor, the artist, by drawing directly upon the lithographic stone or the metal plate, can now dispense with the services of the engraver, who so often denaturalizes the artist's work. Mr. Bellet, encouraged by his first success, has taken out patents in every country, and a company has been formed which will be able to provide a series of apparatus by which a person wholly ignorant of the properties of electricity can reproduce almost instantaneously the most delicate and complicated designs. By the means of slight modifications the apparatus will produce pounce patterns similar to those obtained by Edison's pen, lithographs on stone, etchings, and stereotype plates. The inventor imagines that this invention will lead to a revolution in the manner of illustrating books and papers.—*Journal of Chemistry.*

**A Princely Trial of an old Remedy.**—Under the heading, "The Prince of Wales's Courage," the London World relates the following incident: "The heir apparent and Dr. Lyon Playfair were standing near a caldron containing lead which was boiling at white heat. 'Has your Royal Highness any faith in science?' said the doctor. 'Certainly,' replied the prince. 'Will you, then, place your hand in the boiling metal and ladle out a portion of it?' 'Do you tell me to do this?' asked the prince. 'I do,' replied the doctor. The prince then ladled out some of the boiling lead with his hand, without sustaining any injury. It is a well-known scientific fact that the human hand may be placed uninjured in lead boiling at white heat, being protected from any harm by the moisture of the skin. Should the lead be at a perceptibly lower temperature, the effect need not be described. After this, let no one underrate the courage of the Prince of Wales." There seems a spice of flunkeyism in this. The prince may be supposed to have learned when he was a schoolboy that the experiment is perfectly safe. It requires a little "nerve," to be sure, to perform it, but can hardly be considered a test of one's "courage." *Journal of Chemistry.*

**A Substitute for the Horse.**—A number of country practitioners in England are employing bicycles or tricycles as a means of locomotion, and the use of these vehicles is increasing considerably. They do not supply the place of a horse entirely, but they enable physicians to do away with an extra one. The bicycles are made of iron and steel, the rim of the wheel being covered with rubber. Upon them one can travel over tolerably rough and icy roads, and up quite steep grades. On good ground the rate of speed is a mile in five minutes; racing speed being, however, much greater. The ordinary rate of travel is eight or ten miles an hour. Tricycles are also made, which are safer than the bicycles and nearly as fast. In these the rider sits between two wheels which he propels by a treading motion; a third and guiding wheel is placed in front. There are very likely many places in this country where this mode of locomotion could be used with advantage.—*Hospital Gazette.*

**A Scientific Hoax?**—An improbable story reaches England from Australia concerning an invention by a Signor Rotura. Life is temporarily suspended and restored at pleasure by the use of poison and its antidote, and the interval is indefinitely prolonged by cooling the body, meanwhile, below the lowest temperature at which mortification or putrefaction could take place. A dog, a sheep, an ox, may thus be rendered cataleptic, preserved, and after weeks or months restored to life. Thus they will be packed in the cold hold of the ship at Sidney, and, being restored to life at Liverpool, will walk off to an English market. This "wonderful discovery" has been the theme of much comment in the English papers, and has led to an interesting address on the subject of "suspended animation" by Dr. B. W. Richardson, who shows that, improbable as the alleged invention may be, it is by no means outside the range of scientific possibilities.—*Journal of Chemistry.*

**Physiological Action of Borax.**—It is well known that borax has been advantageously applied in preservation of meat. Some experiments have lately been made by M. de Coyon as to the physiological action of that substance. He fed dogs by one series of experiments, on meat preserved by M. Jourde's process, and in another on fresh food to which various quantities of borax were added. It was found that borax added to meat to the extent of 12 grammes daily (which is ten times what the Jourde's process requires), may be taken in diet without causing the least disorder of general nutrition. Further, borax substituted for common salt increases the power of assimilating meat, and may greatly increase the weight of an animal, even when the alimentation is exclusively albuminoid. These observations, we are reminded, apply only to pure borax, that is, containing neither salts of alum and lead nor carbonate of soda, which are often met with in the borax of commerce.

THE new sphygmophone, invented by Dr. Benjamin W. Richardson, makes the movements of the arterial pulse so audible that the sounds can be distinctly heard throughout a room large enough to hold more than a hundred people. The apparatus consists of an ordinary instrument for the registration of the pulse, with magnetic and telephonic attachments. This invention suggests the possibility that a physician in his office might hear the heart-beats of his patient a mile distant. Dr. Richardson traces a strong resemblance to the words "bother it" in the sound of the human pulse under natural conditions.

